# Is radiation damage the limiting factor in high-resolution single particle imaging with X-ray free-electron lasers?

**DOI:** 10.1063/1.5098309

**Published:** 2019-08-20

**Authors:** C. Östlin, N. Timneanu, C. Caleman, A. V. Martin

**Affiliations:** 1 Molecular and Condensed Matter Physics, Department of Physics and Astronomy, Uppsala University, Box 516, SE-751 20 Uppsala, Sweden; 2 School of Science, RMIT University, Melbourne, Victoria 3000, Australia

## Abstract

The prospect of single particle imaging with atomic resolution is one of the scientific drivers for the development of X-ray free-electron lasers. The assumption since the beginning has been that damage to the sample caused by intense X-ray pulses is one of the limiting factors for achieving subnanometer X-ray imaging of single particles and that X-ray pulses need to be as short as possible. Based on the molecular dynamics simulations of proteins in X-ray fields of various durations (5 fs, 25 fs, and 50 fs), we show that the noise in the diffracted signal caused by radiation damage is less than what can be expected from other sources, such as sample inhomogeneity and X-ray shot-to-shot variations. These findings show a different aspect of the feasibility of high-resolution single particle imaging using free-electron lasers, where employing X-ray pulses of longer durations could still provide a useful diffraction signal above the noise due to the Coulomb explosion.

## I. INTRODUCTION

X-ray crystallography has so far proven to be the most successful technique for determining three-dimensional molecular structures at atomic resolution and has therefore been instrumental in a number of scientific fields. In particular, studies of biological focus have seen a multitude of breakthroughs directly linked to this method. Illuminating a crystalline sample with X-ray radiation and collecting the resulting diffraction patterns have allowed for the structural determination of over 120 000 biomolecules to date, and the number of entries in the Protein Data Bank (PBD)^1^ keeps growing every year.

In conventional, synchrotron-based X-ray crystallography, the periodic structure that makes up the crystal amplifies the diffracted signal and results in an interference pattern with sharp peaks in intensity. These so-called Bragg spots encode for the desired structural information, and their brightness is highly dependent on the size of the crystal. However, the process of crystallizing a biologically relevant molecule is an intricate one; many proteins form inadequately sized crystals, while others do not crystallize at all. With a decreased crystal size, the Bragg spots become less distinct and structure retrieval may become compromised. When using a single molecule with no periodicity, even more signal is lost, as the constructive interference of the outgoing waves no longer produces Bragg spots. The only usable features for structure determination that remain, often referred to as speckles, arise from interfering waves from different atoms within the same molecule. In this case, a different approach is necessary.

Cryo-electron microscopy (cryo-EM) of single particles has recently seen some significant improvements in terms of resolution and holds considerable promise.^2^ Important structures such as ribosomes, membrane proteins, and hemoglobin have been solved to near-atomic resolution using this method.^3–5^ As a nondiffractive technique, it bypasses some of the challenges facing X-ray-based setups, but its applicability to smaller samples such as individual proteins is still limited and faces other types of challenges.

Another aspiring method is to employ the high-intensity light pulses offered by X-ray free-electron lasers (XFELs) to enhance the weak signal. However, this leads to severe radiation damage and ultimately an explosion of the small sample.^6–8^ The ionization and subsequent destruction of the sample limit the achievable resolution in two ways. The loss of bound electrons causes the system to scatter less X-rays, and the structural changes caused by the Coulomb forces destroy the native structure of the sample, adding noise in the diffraction pattern. While this has been overcome with pulse durations short enough to outrun most of the damage processes for nanometer-sized crystals in serial femtosecond crystallography experiments,^9–12^ the realization of atomic-resolution single particle imaging (SPI) remains challenging. The ionization and decay of the molecule during exposure cause noise in the diffraction image, which limits the resolution of the reconstructed image. Pulses as short as 5 fs have been suggested to be necessary for the successful imaging of an undamaged molecule to atomic resolution.^13^ Schemes for generating such short pulses have been proposed,^14^ and there is hope that higher intensities could be made available in the near future.

It is worth noting that our question of radiation damage limits concerns the ultimate goal of imaging of single proteins at high resolutions, i.e., <3 Å, with comparable results to crystallography and cryo-EM. These are the resolutions required to fit atomic models to the electron density and impact structural biology. XFEL single particle imaging is currently producing images of viruses with the order of few nanometers resolution. We do not suggest that radiation damage is the limiting factor for current SPI experiments at these lower resolutions, which is likely due to the experimental challenges of background scattering, sample injection, data collection rates, and other technical challenges outlined in the SPI roadmap.^15^ Instead, our aim is to contribute to the understanding of theoretical limits of SPI at high resolution which the field ultimately aspires to reach in the longer term.

It has previously been shown that the loss of structural coherence in nanocrystals provides a gating effect for the Bragg diffraction, essentially enabling imaging with longer pulses.^16^ A similar idea has been investigated for SPI by simulating damage noise as a consequence of ionization and spatially uncorrelated ion diffusion.^17^ The study found a gating effect due to damage analogous to the crystalline case but recognized the limitations in omitting the influence of the Coulomb explosion. Here, we aimed to complement this by applying an equivalent methodology to simulations of an exploding molecule. Building on our recent study of the explosion dynamics of proteins exposed to an XFEL pulse,^8^ we investigated how the Coulomb explosion contributes to the noise in the diffracted signal. The impact was evaluated through comparisons to noise caused by structural variations within the sample, i.e., sample heterogeneity, and due to deviations in scattering between pulses, the so-called shot noise.

To develop SPI toward higher resolution imaging than is achievable today, it will be important to understand how the explosion influences the speckle contrast seen in the diffraction patterns. This has the potential to refine the orientation recovery further, thereby reducing the number of diffraction patterns needed to generate a complete 3D dataset^18^ The development of this theoretical framework also allows for optimizations of structure reconstruction algorithms.

## II. METHOD

### A. Explosion simulations

Following our earlier work,^8,19,20^ we simulated the interaction between a lysozyme protein molecule (PDB identifier 1LYS^21^) and an ultrashort XFEL pulse using the molecular dynamics (MD) software GROMACS 3.^22^ MD is a simulation scheme based entirely upon classical mechanics that has been shown to accurately describe such interactions.^23^ We note that lysozyme is small compared to SPI samples in current experiments, but it was not feasible to simulate heterogeneity and damage in a larger sample with MD over a range of pulse parameters with current computational resources. Simulating a smaller sample is consistent with other MD damage studies of SPI.^24^ The force field employed was the well-known all-atom Optimized Potentials for Liquid Simulations (OPLS-AA),^25^ and bonds were modeled with Morse potentials to enable bond-breaking. Six sets of pulse parameters were chosen in accordance with those available at a typical XFEL biomolecular imaging beamline, such as CXI^26^ at the Linac Coherent Light Source (LCLS), SLAC National Accelerator Laboratory. Photon energies and focal spot diameters were kept constant over the sets at 8 keV and 100 nm, respectively, while intensities and pulse durations were changed. The Gaussian temporal profile pulses were given three different FWHM durations of 5, 25, and 50 fs, each with either 10^12^ or 10^13^ photons in total. Each sample was placed in the center of the focal spot, over which the photons were uniformly distributed, and therefore fully immersed in the beam. These pulse conditions with 10^12^ photons per pulse are close to what is available at current XFEL facilities. The case of 10^13^ photons is one order of magnitude more intense than currently available but shows how an advancement in XFEL intensity impacts radiation damage.

The simulations were carried out using the GROMACS function “ionize” with stochastic ionization sequences, as described by Neutze *et al.*^6,27^ In their work, they refer to the ionizing function as XMD. For each set of pulse parameters, a total of *N* = 150 explosions were simulated with samples in the same spatial orientation. Initial structures were selected at random from a separate vacuum simulation at room temperature, generating slight structural variations to emulate sample heterogeneity. By comparison, the mean root mean square deviation value based on all atoms significantly contributing to scattering (C, N, O, and S) between all chosen structures was measured to be 0.95 ± 0.1 Å. The total simulation time was 200 fs and employed 50 as time steps, with frames specifying the atomic positions being collected every 0.5 fs. The ionization states of all atoms were also extracted, yielding a set of “ionization frames” complementary to the positional frames.

The ionize function in GROMACS considers three main types of ionization processes—photoionization of core electrons, photoionization of valence electrons, and Auger decay—and monitors these throughout the simulation. An atom or ion is modeled to have a maximum of two core electrons, and the rest is allotted to the valence shell. Ionization cross sections for the two shells in different atomic species are based on experimental data at various photon energies, but the code also allows for interpolation to unlisted energies. Secondary processes such as electron collisions are disregarded, but while generally not applicable to a conventional crystallographic setup with large crystals, we assume this approximation to be acceptable here due to the small sample size and shorter pulses.^19^

We have further employed a multidimensional nonlocal thermodynamic equilibrium (nLTE) radiation transfer code to calculate the ionization in an infinite sample containing lysozyme. This was done to compare the ionization in the single protein case, using GROMACS, to a model with a more robust ionization treatment and a solid sample—where we expect the electron impact ionization to play a more pronounced role. The nLTE model is based on a plasma description and implemented in the code CRETIN.^28^ The code has been used to describe the interaction between XFEL pulses and biological matter at several occasions,^11,12^ and has proven to be able to reproduce experimental results.^10,29^ CRETIN provides the electronic level populations, transmission rates, absorption, heating rates, and conduction coefficients for each time step of the simulation. The simulations presented in the present study are performed in the same way as in our earlier work,^30^ but with the number of photons per pulse and the photon energy similar to what we used in the MD simulations. We refer the reader our earlier work, mentioned above, and references therein for the details of the simulations.

### B. Diffraction patterns

The MD-trajectories were used as a basis for the calculation of diffraction patterns. The simulated detector had a pixel count of 256 × 256 with each pixel being 33.8 *μ*m wide. The detector was placed at a distance of 3 mm from the sample, suitable for the analysis of resolutions from ∼15 Å to ∼1.46 Å at the corner of the detector for 8 keV X-rays. Current SPI experiments are pushing toward 1 nm resolution, while the future goal of 3 Å resolution would enable molecular structure determination comparable to crystallography or cryo-EM. We have chosen a resolution range to span these target resolutions, although we note that our maximum simulated resolution of 1.46 Å is beyond what is reasonably expected to be achieved. This detector geometry can easily be scaled up with a numerical factor to better correspond to experimental conditions. For example, using a factor 10 would give a distance of 30 mm from the sample to the detector, and the detector could have 1024 × 1024 pixels of size 84.5 *μ*m, which can be seen as 256 “effective pixels” for analysis purposes.

A diffraction pattern was calculated from each frame of the explosion simulation, separated in time by 0.5 fs, giving a set of instantaneous, noiseless patterns reflecting the full time evolution of the molecule during X-ray exposure. The code used was developed by Martin,^17^ but expanded on here to allow for the inclusion of ionization data generated by GROMACS. In the code, a diffraction pattern is calculated as

$$I_{n}(q,t_{f})=r_{e}^{2}P(q)d\Omega I(t_{f})$$

$$\times \left[\sum_{i=1}^{M}A_{i}(q,t_{f})+2\sum_{i=1}^{M}\sum_{j=1}^{i-1}B_{\mathrm{ij}}(q,t_{f})\right],$$ (1)

where **q** is the scattering vector, *t_f_* is the time stamp of frame *f*, *r_e_* is the classical electron radius, *P*(**q**) is a polarization term, *d*Ω is the solid-angle, and *M* is the total number of atoms in the sample. Finally, the index *n* = 1, 2,…, *N* indicate the explosion event. Note that in our previous work,^17^ the diffraction contribution of sulfur was ignored, but in this work, we include all elements of the sample in the diffraction calculations.

*I*(*t_f_*) is the incident X-ray intensity, and since GROMACS simulates an X-ray pulse with normally distributed photons in time, it is time dependent. To reflect this in the generation of diffraction patterns, *I*(*t_f_*) is calculated separately for each time frame analyzed. The Gaussian describing the photon pulse is defined as

$$G(t)=\frac{2N_{p}}{T}\sqrt{\frac{\;\text{ln}\;2}{\pi }}e^{-4\;\text{ln}\;(2)\frac{t^{2}}{T^{2}}},$$ (2)

where *N_p_* is the total number of photons and *T* is the FWHM duration of the pulse. As such, it is normalized to the total number of photons in the pulse

$$\int_{-\infty }^{+\infty }G(t)\mathrm{dt}=N_{p}.$$ (3)

The evaluated time frames are separated in time by Δ*t* = 0.5 fs, so to determine the incoming intensity in each time step, we numerically integrate *G*(*t*) in a discretized fashion.

The terms in square brackets of Eq. (1) are given by the atomic scattering factors *f_i_*(*q*, *t*) and positional vector **R**_*i*_ of each atom *i*,

$$A_{i}(q,t_{f})=|f_{i}(q,t_{f}){|}^{2},$$ (4)

$$B_{\mathrm{ij}}(q,t_{f})=f_{i}(q,t_{f})f_{j}(q,t_{f})\;\text{cos}\;\left\{2\pi q(R_{i}(t_{f})-R_{j}(t_{f}))\right\}.$$ (5)

The electron density, and by extension the form factor, of an atom is assumed to be spherically symmetric, which therefore is written as a function of the magnitude of the scattering vector, q=|q|. In our calculations, *q* translates to spatial resolution as 1/*q*. Moreover, *f_i_*(*q*, *t*) changes with the time-dependent ionization levels, which is accounted for here. Slater orbitals are used to calculate the ionic scattering factors in any given frame based on the ionization events determined by GROMACS.^31^

Once the full set of patterns over an explosion event had been calculated, they were added to form a representation of the integrated pattern measured in an SPI experiment,

$$I_{n}(q)=\sum_{f}I_{n}(q,t_{f}).$$ (6)

We refer to these as “time-integrated” patterns.

### C. Analysis

**FIG. 1.**
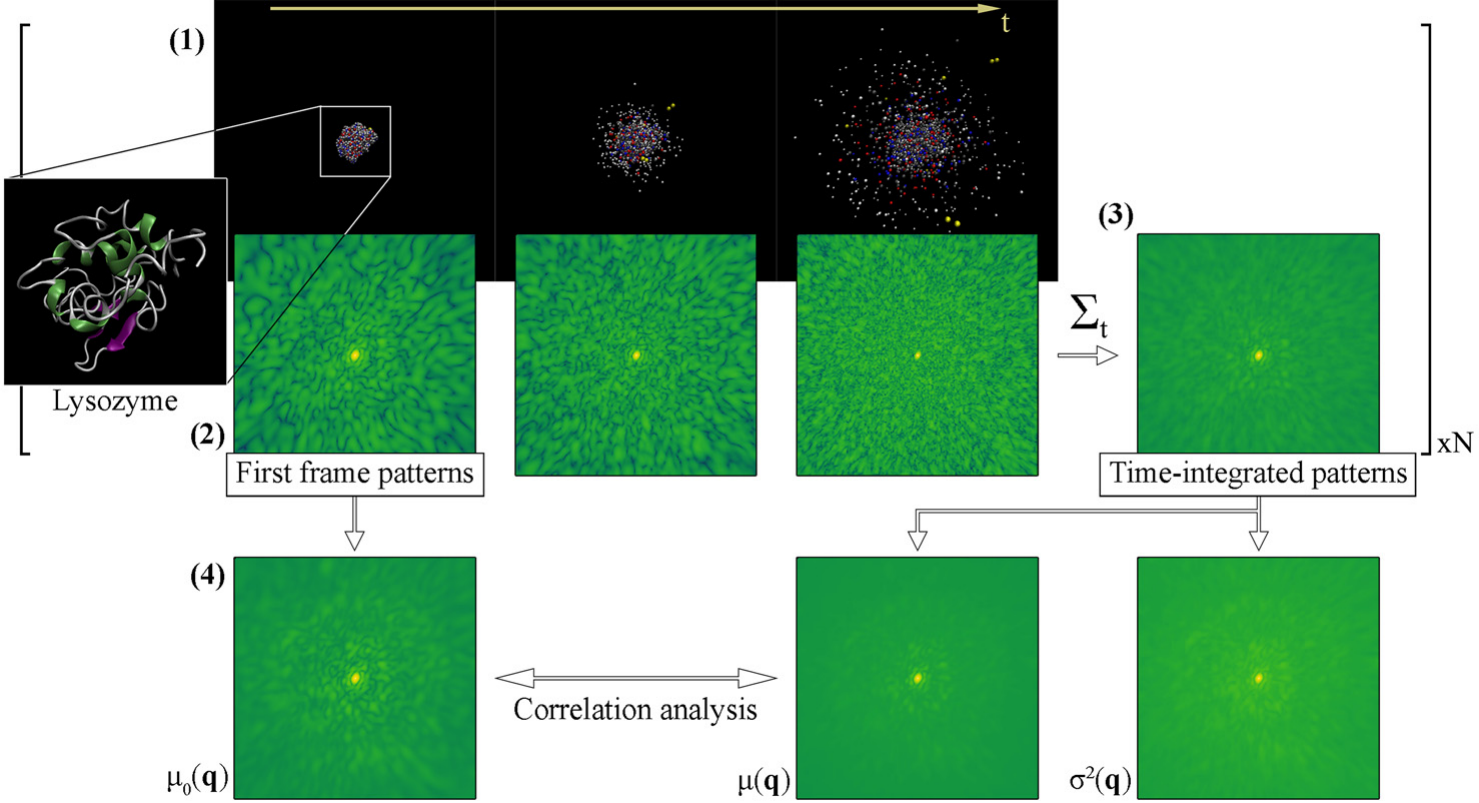
Illustration of data collection and analysis. (1) Molecular dynamics simulations predict the time evolution of a single lysozyme molecule while exposed to an XFEL pulse. A number of *N* = 150 simulations were performed for every set of pulse parameters with heterogeneous starting structures and stochastic ionization. Note that only three frames are shown here. (2) Diffraction patterns are calculated from the individual frames of the simulations, generating a time series of instantaneous snapshots for each explosion event. (3) Each series is summed incoherently to produce a time-integrated pattern akin to the measured diffraction pattern from a single pulse in a SPI experiment. (4) Mean pixel intensities over all explosions within the same pulse parameter set are calculated for the first frame patterns and time-integrated patterns. The former holds the undamaged structural information—slightly blurred due to sample heterogeneity—while the latter is affected by damage noise. These two are compared through Pearson correlation analysis to ensure the validity of the observed features, and the pixel variance of the time-integrated patterns is calculated to gauge damage-induced information loss.

The following analytical pipeline, illustrated in Fig. 1, was applied to each of the different pulse parameter sets. Time-integrated patterns of all *N* explosions were first used to calculate two aggregate 2D maps. The expected intensity

$$\mu (q)=\frac{1}{N}\sum_{n=1}^{N}I_{n}(q)$$ (7)

shows the mean accumulated signal in each detector pixel over all explosions. This can be considered the statistically average pattern measured in a SPI experiment of the given pulse specifications and sample orientation. Usually, the imaging problem is formulated with the goal of recovering a merged 3D intensity equal to the square of the molecular scattering factor. The aim of an orientation algorithm is to produce a merged 3D intensity as close as possible to that goal in the presence of experimental noise. If damage is present, then the square of the undamaged molecular scattering factor is not a realistic goal. An orientation algorithm can at best aim to reproduce *μ*(**q**).

In SPI experiments, we expect average patterns to be used when the scattering is sparse, as well as in the procedure of 3D assembly of patterns for structural determination. The quality of the averaged patterns will then be highly dependent on the variations between the individual patterns measured. For this reason, we also calculate the pixel variance between integrated patterns

$$\sigma^{2}(q)=\frac{1}{N}\sum_{n=1}^{N}{\left(I_{n}(q)-\mu (q)\right)}^{2},$$ (8)

which gives insights into the fluctuations of measured intensities in a given pixel for different damage scenarios.

Because the time-integrated patterns are blurred due to noise caused by ionization and atomic displacement, the quality of the structural information within them is uncertain. To ensure the reliability of the speckles in the average accumulated signal, we compare it to the undamaged case by Pearson correlation. The Pearson correlation is a widely used measure of similarity between two functions adjusting for the mean and overall normalization. It has been shown to be a successful metric for use in orientation algorithms.^32^ We define the undamaged pattern as the average first-frame diffraction pattern of all simulations

$$\mu_{0}(q)=\frac{1}{N}\sum_{n=1}^{N}I_{n}(q,t_{0}),$$ (9)

similar to the average time-integrated pattern. Both of these patterns show a bright central speckle up to a scattering length of *q* ≈ 0.5 nm^−1^ (corresponding to 20 Å resolution) that is not measured experimentally since it overlaps with the unscattered beam. To mask the speckle out in the correlation analysis, we dismiss all detector pixels at $q\leq 0.65\;{\text{nm}}^{-1}$, which translates to real space distances of >15 Å. We let $\tilde{q}=\{q:q>0.65\;{\text{nm}}^{-1}\}$ and calculate the pixelwise Pearson correlation coefficient as

$$r=\frac{\sum_{\tilde{q}}\left(\mu (\tilde{q})-\langle \mu (\tilde{q})\rangle \right)\left(\mu_{0}(\tilde{q})-\langle \mu_{0}(\tilde{q})\rangle \right)}{\sqrt{\sum_{\tilde{q}}{\left(\mu (\tilde{q})-\langle \mu (\tilde{q})\rangle \right)}^{2}}\sqrt{\sum_{\tilde{q}}{\left(\mu_{0}(\tilde{q})-\langle \mu_{0}(\tilde{q})\rangle \right)}^{2}}}.$$ (10)

Here, ⟨·⟩ denote mean values. The possible values of *r* range from –1 to 1, with 0 indicating no correlation between the patterns, and –1 and 1 corresponding to perfect negative and positive correlations, respectively. As such, for structural information to be retrievable and accurate in the time-integrated pattern, values closer to 1 are necessary.

The term *r* measures the isolated effects of damage noise, and to some extent sample heterogeneity, on the measured diffraction pattern. The latter is mostly suppressed due to the averaging over a set of structural variations, so any observed deviations from perfect correlation are the results of damage. By comparison, two other sets of correlation coefficients were calculated at each pulse intensity. First, we correlated the noiseless first-frame patterns from varying starting structures to capture the effects of sample heterogeneity. Pairs of patterns were randomly selected from the full collection of 450 starting structures and correlated, and the process was iterated 50,000 times. Second, an instantaneous noiseless pattern was calculated at full intensity from one of the 450 structures, chosen at random. Shot noise was added independently to the pattern two times by Poisson sampling of the pixels, and the noisy patterns were correlated. Again, this was repeated 50 000 times. These datasets capture the effects of sample heterogeneity and shot noise on the correlation coefficient, respectively, and allow us to determine the severity of damage noise in relation to the other noise sources.

The magnitude of the scattering vector encodes for the resolution in real space, which means that speckles correspond to higher resolution structural features the further from the detector center they appear. Each ring of pixels at a set distance from the center therefore contains information of a specific resolution, here referred to as a “resolution shell.” Because the rate of degradation of the diffraction signal likely is resolution-dependent, it is also of interest to investigate the correlation between the first frame and time-integrated patterns at individual resolution shells. Under the assumption that there is no favored directionality present in the sample, we radially integrate over each resolution shell of the maps defined above and obtain one-variable functions of the mean intensity and variance. We let *θ* be the angular component of the scattering vector **q** = (*q*, *θ*) in polar coordinates and define

$$\mu (q)=\frac{1}{2\pi }\int_{0}^{2\pi }\mu (q)d\theta$$ (11)

and

$$\sigma^{2}(q)=\frac{1}{2\pi }\int_{0}^{2\pi }\sigma^{2}(q)d\theta .$$ (12)

The same integration scheme is applied to the undamaged case *μ*_0_(**q**), allowing us to formulate the Pearson correlation coefficient as a function of *q*,

$$r(q)=\frac{\sum_{\theta }\left(\mu (q,\theta )-\mu (q)\right)\left(\mu_{0}(q,\theta )-\mu_{0}(q)\right)}{\sqrt{\sum_{\theta }{\left(\mu (q,\theta )-\mu (q)\right)}^{2}}\sqrt{\sum_{\theta }{\left(\mu_{0}(q,\theta )-\mu_{0}(q)\right)}^{2}}}.$$ (13)

The results of the resolution-dependent correlations are analyzed in a similar manner to their pixelwise counterparts. We also include resolution shell correlations of patterns from heterogeneous samples and patterns with shot noise for comparison as before. Note that in this case we get a unique distribution of correlation values for every resolution shell. From these, we extract the mean values and standard deviations, providing the graphs with appropriate error bars.

Next, we define the speckle contrast as the standard deviation within each resolution shell

$$\sigma_{I}(q)=\sqrt{\frac{1}{2\pi }\int_{0}^{2\pi }\mu^{2}(q)d\theta -\mu^{2}(q)},$$ (14)

which essentially describes the signal we are interested in for imaging purposes. In order for structural determination to be feasible, the speckles must display sufficiently high contrast to be distinguishable from noise.

The noise considered here stems from two sources: shot-to-shot fluctuations and changing diffraction conditions throughout the pulse due to sample damage. The mean intensity function [Eq. (11)] allows for the estimation of the former, which is a Poisson process, as $\sqrt{\mu (q)}$. We refer to this measure as the “shot noise.” The second source of signal masking is a direct consequence of the Coulomb explosion that the molecule undergoes. The ionization and displacement of the target atoms affect the diffraction signal such that the pattern generated in each time step deviates from the previous one. The behavior is captured by the variance function [Eq. (12)], so we define the “damage noise” as the standard deviation *σ*(*q*).

With these quantities in place, we examine various signal-to-noise ratios (SNRs) to evaluate the possibilities of successfully imaging the sample molecule under the given conditions. *SNR_S_* (shot noise) and *SNR_D_* (damage noise) show how the two different sources of noise affect the signal separately,

$$\mathrm{SN}R_{S}(q)=\frac{\sigma_{I}(q)}{\sqrt{\mu (q)}},$$ (15)

$$\mathrm{SN}R_{D}(q)=\frac{\sigma_{I}(q)}{\sigma (q)}.$$ (16)

This is important since the main obstacle for imaging with longer X-ray pulses is believed to be the additional ionization and consequent deterioration of the structure.

A complete picture is obtained by comparing the calculated signal to both noise contributions simultaneously. The signal to total noise ratio, *SNR_tot_*, indicates the levels of signal we ultimately can expect to measure through the noise and can be written as

$$\mathrm{SN}R_{\mathrm{tot}}(q)=\frac{\sigma_{I}(q)}{\sqrt{\mu (q)+\sigma^{2}(q)}}.$$ (17)

In previous studies, it has been reported that both single molecules and crystalline samples may exhibit a so-called self-gating effect.^16,17^ The diffraction signal is predicted to terminate during the pulse due to damage, shortening the effective pulse duration and thus enabling imaging with longer X-ray pulses. To evaluate if such an effect also is observed when modeling the Coulomb explosion, we look into the evolution of *SNR_tot_* throughout the exposure. By partially integrating the series of instantaneous diffraction patterns in time and applying the described analysis pipeline, we get SNR-values accumulated up to different time steps. In the case of self-gating, we would expect a saturation point to be reached after which *SNR_tot_* remains constant. In addition to the patterns analyzed so far, where the combined effects of sample ionization and atomic displacement are included, each time step was complemented with two analogous calculations of diffraction patterns. One of them considered ionization as the only contribution to damage by fixing the initial atomic positions in space and applying the ionization data as before. Conversely, the other calculation scheme allowed the atoms to move as predicted by GROMACS but disregarded their state of ionization to isolate the effects of atomic displacement. In other words, we performed the same analysis as before (with partial integration of the patterns) but calculated the diffraction patterns from each frame as if all the atoms were in the ground state rather than ionized. The inclusion of these extra datasets lets us not only investigate the existence of a gating effect but also how it is influenced by the two processes.

## III. RESULTS AND DISCUSSION

**FIG. 2.**
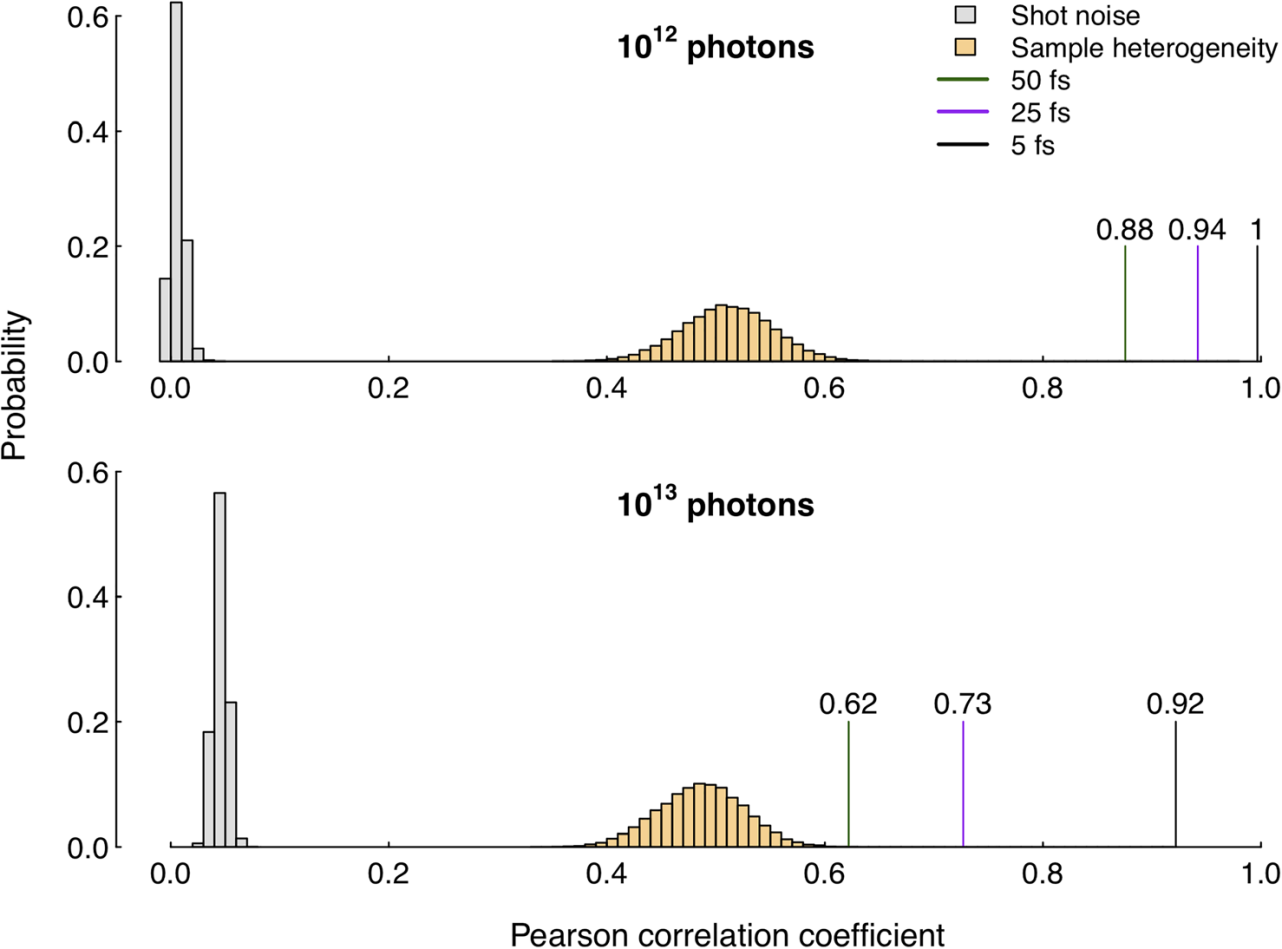
Pixelwise Pearson correlation. The vertical lines highlight the values obtained when correlating the diffraction pattern subject to damage (mean time-integrated) to the undamaged counterpart (mean first-frame) at various pulse conditions. This indicates that high intensities and long pulses lead to greater losses in structural information of the recorded diffraction pattern due to damage. However, even at the most unfavorable parameters investigated, the similarity to the undamaged case is still high and remains above the corresponding values when correlating patterns from heterogeneous samples (orange bars) or patterns affected by shot-to-shot fluctuations (gray bars). The correlation values all have a statistical significance with p-values ≈0 for all pulse parameters due to the large number of detector pixels.

The pixelwise correlation analysis shows good to excellent positive conformity (correlations values > 0.6) between the average first frame and time-integrated patterns for all parameter sets, as indicated in Fig. 2. Keeping the limited number of data points in mind, correlation values seem to decrease with longer pulse durations and the decrease happens more rapidly at higher pulse intensities. This is to be expected as longer exposure times allow for sample damage to manifest to a greater extent, while higher intensities will promote the onset of damage processes more efficiently. Both a larger number of time frames and a more rapid modification of the sample force greater variations between diffraction patterns. The average time-integrated pattern will therefore deviate more from the mean first frame pattern than their low intensity, short duration counterparts. However, all values stay well above the comparative threshold values from sample heterogeneity and shot noise, which indicates that the speckles correspond well to the initial structure and its specific orientation.

**FIG. 3.**
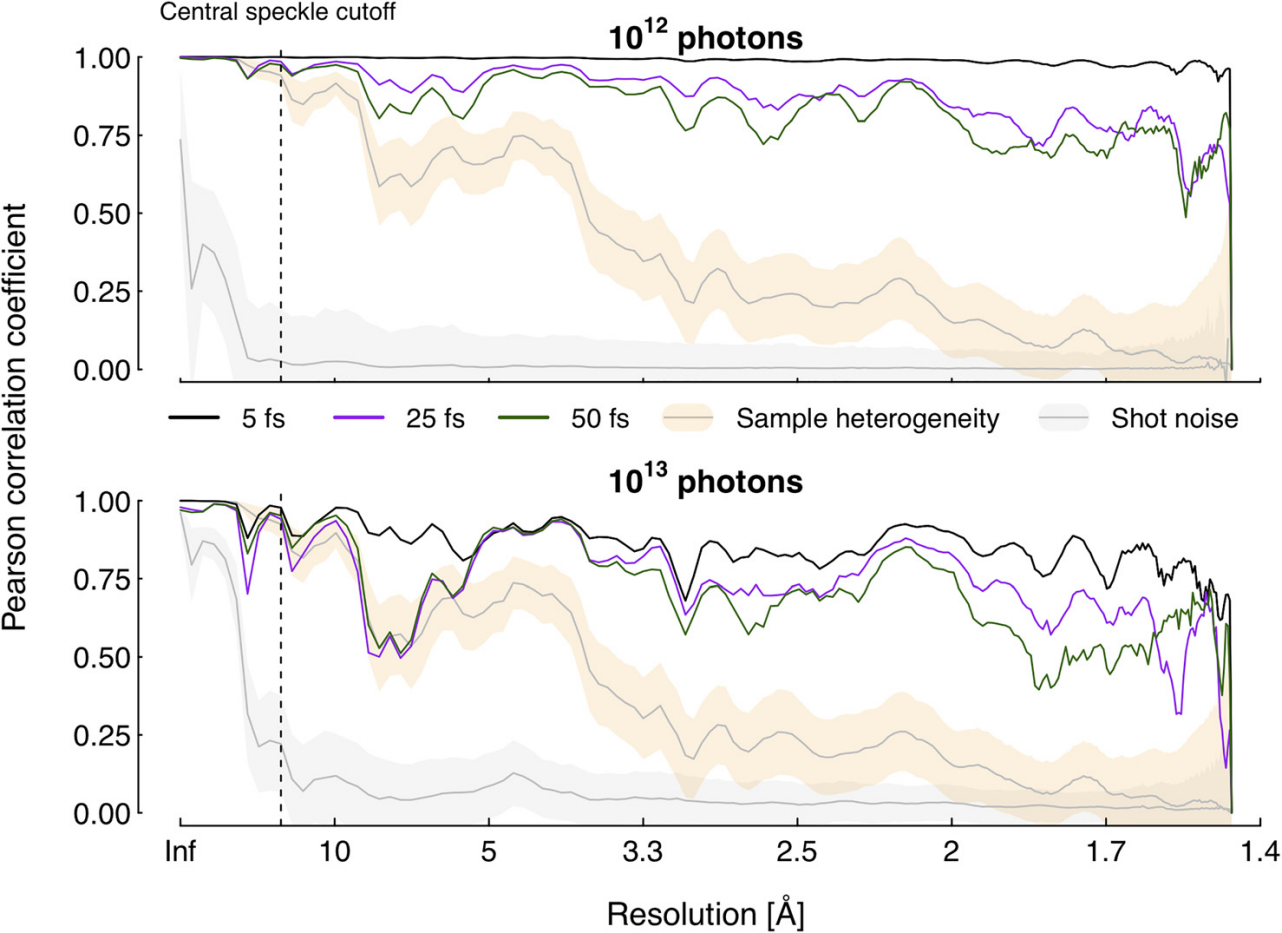
Resolution-dependent Pearson correlation. Plots show the calculated Pearson correlation coefficient as a function of *q* for different pulse conditions. For comparison, the corresponding mean values when correlating undamaged, noiseless diffraction patterns of heterogeneous lysozyme, as well as a single-structure pattern subjected to shot noise, are shown as gray lines. The shaded orange (sample heterogeneity) and gray (shot noise) areas indicate one standard deviation of the spread above and below their respective mean. The dashed line marks the central speckle cutoff to the left of which data points are not applicable to an experimental setting. The correlation of the time-integrated pattern to the undamaged one remains high throughout all resolution shells, in particular for the lower intensity pulses. With a 10^12^ photon pulse, damage effects bring about a smaller drop in correlation than both sample heterogeneity and shot noise over the entire relevant resolution span. At medium to high resolutions, this also holds true for higher intensity.

Investigating the signal correlation over the resolution shells gives a more detailed picture of the fidelity of the recorded patterns. Figure 3 shows that the *r*-value given by Eq. (13) is consistently high for the lower pulse intensity, not dropping below the threshold values regardless of pulse duration. For the higher intensity, while less consistent, the correlation to the undamaged pattern remains strong—in particular for the shortest pulse. It is therefore reasonable to assume that speckles displayed in the time-integrated patterns correspond well to those generated by the desired structure, even at high resolutions. Moreover, both sample heterogeneity and shot noise seem to blur the wanted speckle features to a far greater extent than damage processes.

It is worth mentioning that since each resolution shell consists of a subset of all detector pixels, the degrees of freedom in the correlation analysis of the individual shells will be lower than that when considering the full detector. This decrease affects the statistical significance of the correlation coefficient negatively, especially near the detector center and edges—i.e., at the limits of low and high resolutions. However, while the effect is particularly impactful at high-resolution scattering, where both pixel and photon counts are low, p-values remain <0.05 up to a resolution of 1.5 Å for all pulse conditions.

**FIG. 4.**
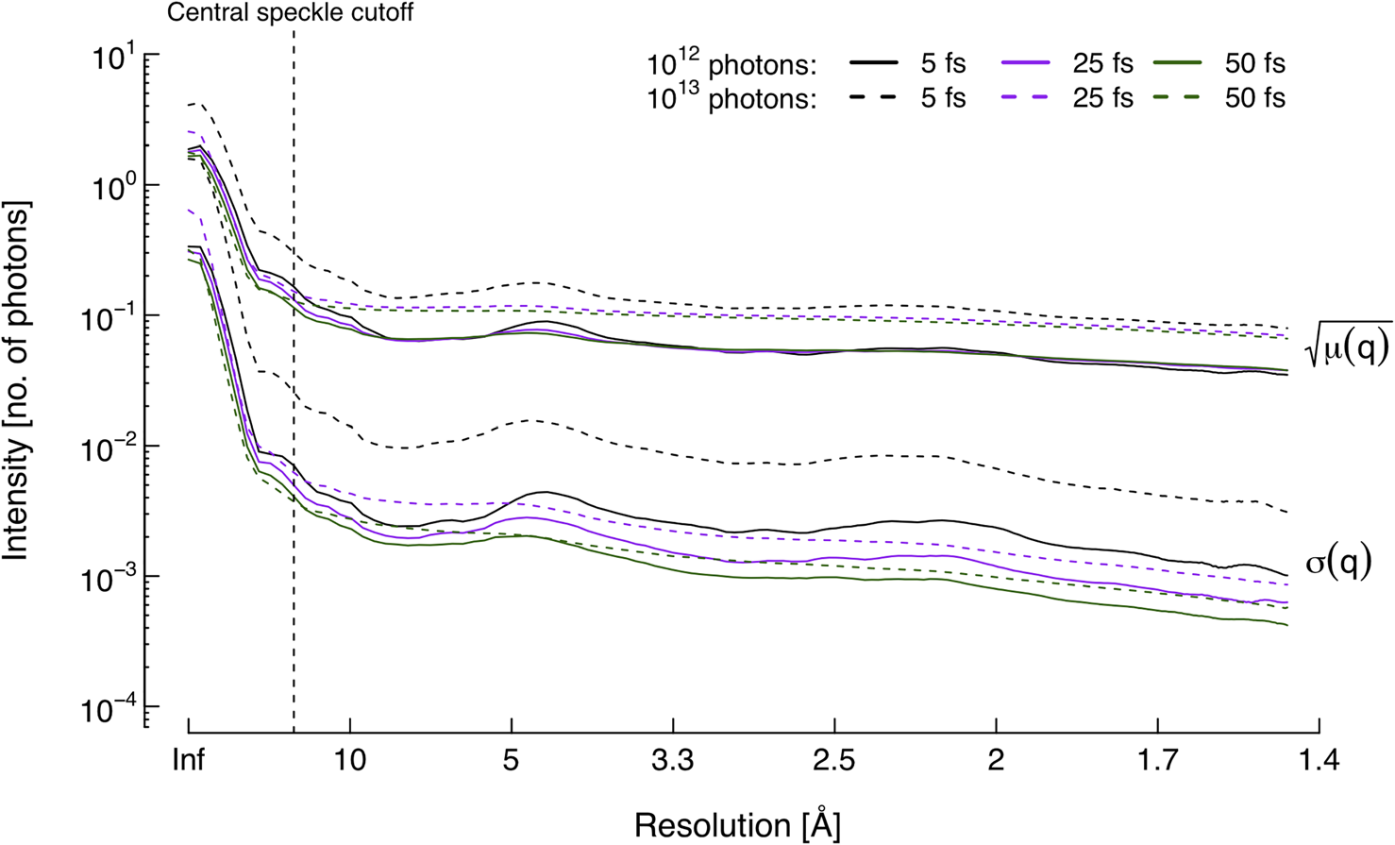
Noise intensities at pulse termination. The shot noise $\sqrt{\mu (q)}$ is clearly the dominant source of signal blurring with intensities at least one order of magnitude higher than damage noise *σ*(*q*) in resolution shells outside of the central speckle. The two noise sources also display dissimilar responses to changes in X-ray pulse conditions, with damage noise being considerably more sensitive to pulse duration.

With the reliability of the integrated signal established, it becomes relevant to assess the signal strength and noise levels in the integrated diffraction patterns. Figure 4 shows the shot noise $\sqrt{\mu (q)}$ and damage noise *σ*(*q*) calculated from the time-integrated patterns [see Eqs. (11) and (12)]. The plots indicate that shot noise dominates damage noise by a factor of at least 10 for resolutions outside of the central speckle cutoff at 15 Å. This is in accordance with a previous study where the effects of the Coulomb explosion were excluded, and a hydrodynamic model was used to estimate damage noise.^17^ The relative low impact of damage is potentially advantageous for imaging; averaging the number of measurements needed to sufficiently suppress shot noise would simultaneously counteract the noise from damage.

Damage noise contributions are related to both pulse duration and intensity. Contrary to what one might expect, a shorter pulse seems to generate more noise due to damage. This is an effect of the higher ionization rate, making the Coulomb explosion less reproducible. The same holds true for the higher intensity pulse, where more photons promote additional sample damage. A similar dependency on intensity is observed for shot noise; shot-to-shot fluctuations are simply greater when the number of scattered photons is higher. However, while damage effects cause a lowering in the scattering cross section during X-ray exposure, the subsequent decrease in shot noise during longer pulses is not as substantial as a 10-fold decrease in the total number of incoming photons. Of the pulse parameters studied here, the duration therefore does not impact the expected shot noise as much as the intensity does. Nonetheless, both sources of noise are minimized with the longest pulse and lower photon flux.

**FIG. 5.**
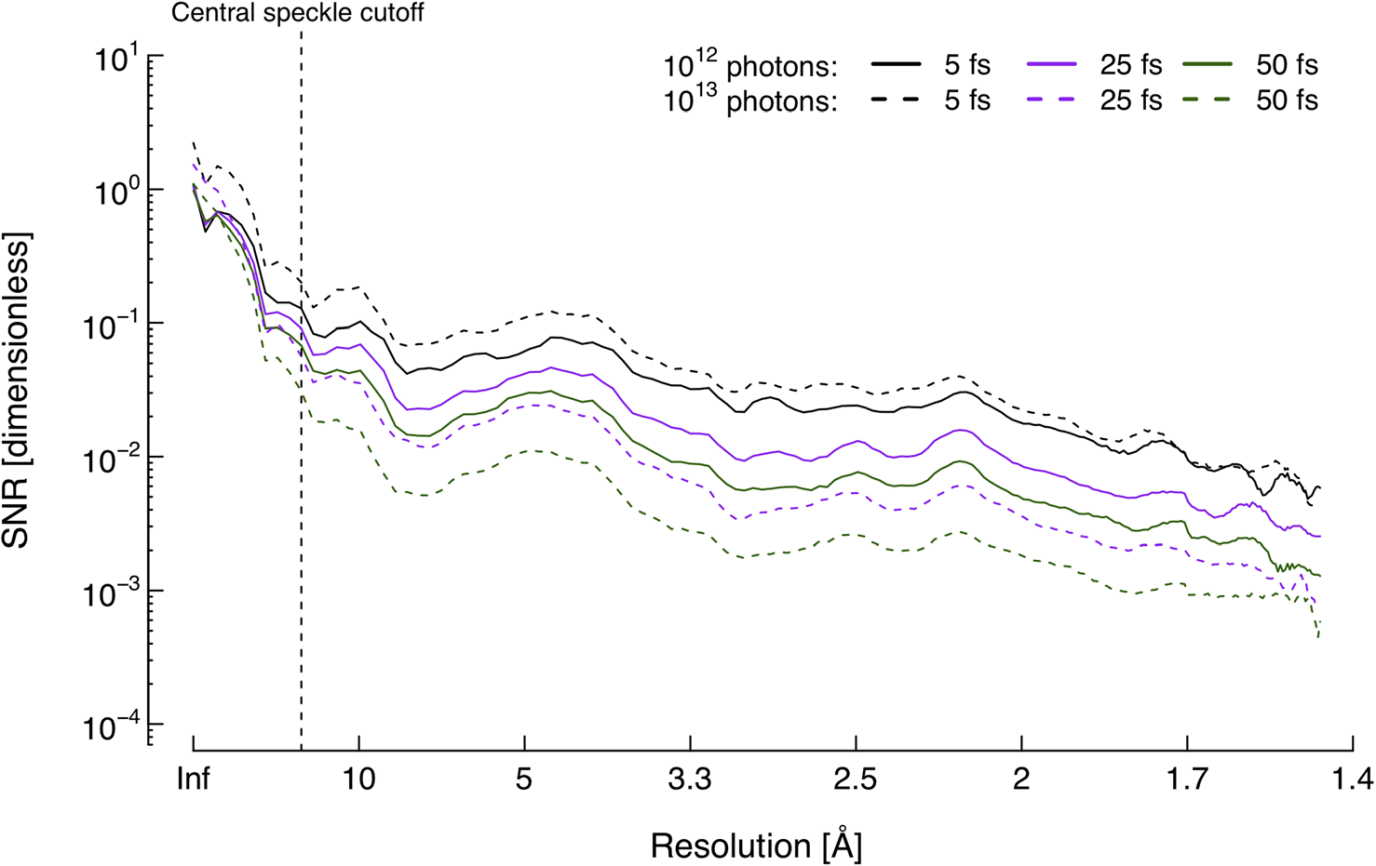
Signal-to-total-noise ratio at pulse termination. Both the pulse duration and intensity affect the resulting *SNR_tot_*, and a trade-off between both parameters to optimize the ratio can clearly be seen. The values were calculated using Eq. (17).

The signal shows similar behavior. Changing the pulse parameters from 5 fs and 10^13^ photons to 50 fs and 10^12^ photons leads to an average 10-fold reduction in speckle contrast over all resolution shells. As can be seen in Fig. 5, despite vaster noise contributions, the SNR is maximized with the shortest pulses since the speckle contrast is enhanced to an even greater extent—especially when the intensity is high. However, while a greater photon flux is preferred when the duration is short, decreasing the number of photons when the exposure time is longer actually improves the quality of the measured diffraction pattern. A lower photon count is beneficial for both 25 and 50 fs pulses compared to their higher-intensity counterparts. Apparently, there is a trade-off between the two parameters, and we conclude that both pulse duration and intensity need to be taken into account concurrently in order to optimize the signal-to-noise ratio. This has important practical consequences since several XFELs currently in operation offer the ability to generate shorter pulses at the expense of pulse intensity.

The signal is significantly weaker at higher resolution, causing the signal-to-noise ratio to drop as we go toward higher scattering angles, despite noise contributions being fairly constant. *SNR_tot_* decreases by more than one order of magnitude between the central speckle (15 Å) and the detector corner (1.5 Å) for all pulses (see Fig. 5). Noise is reduced by the square root of the number of patterns used, so an *n*-fold improvement in SNR requires a factor of *n*^2^ more patterns. At 5 Å resolution, for instance, this means that averaging 16 patterns recorded with 50 fs, 10^12^ photon-pulses would make up for the 4-fold signal-to-noise difference compared to when 5 fs pulses of the same intensity are used.

**FIG. 6.**
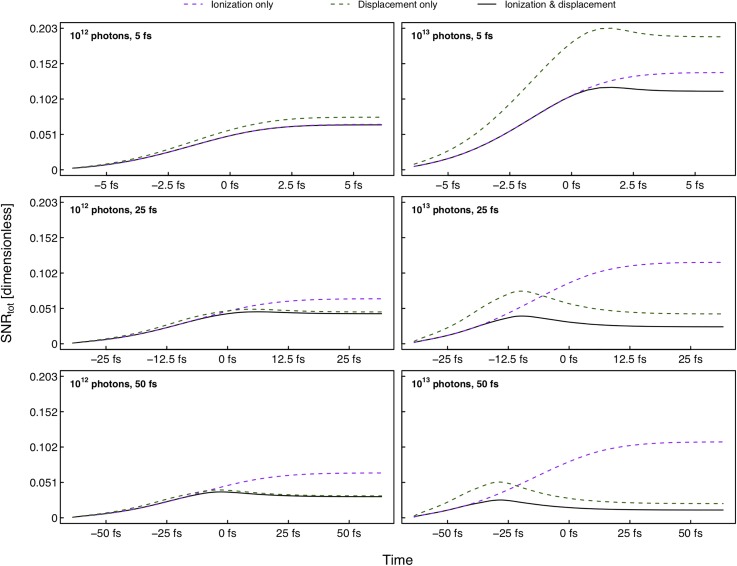
Signal-to-noise accumulation throughout the pulses at 5 Å resolution. The graphs show the time-evolution of *SNR_tot_* when the sample is subjected to ionization (purple), atomic displacement (green), or both (black). The time-dependence is generated by truncating the temporal sum in Eq. (1). A gating effect due to sample damage is observed in all cases, indicating that longer pulses still may be feasible for imaging. The simulated pulses have Gaussian temporal profiles with means at *t* = 0 and FWHM durations as indicated in the top left of each pane.

Equation (1) shows how the diffracted intensity accumulates as a function of pulse time *t_f_*, and by truncating this sum at different time points during the pulse, we can study how derived quantities such as SNR levels behave as a function of pulse time. Looking at the time evolution at scattering angles corresponding to 5 Å resolution, we see that SNR levels accumulate throughout the exposure to reach a point of saturation as shown in Fig. 6 (black lines). The Coulomb explosion causes the accretion of the signal, as well as diffuse scattering contributing to noise, to diminish throughout the pulse duration. Eventually, diffraction is terminated entirely. In some cases, particularly for the longer pulses, the saturation is reached around or even before the bulk of the photons arrive at *t* = 0. Because of the damage onset, trailing photons does not seem to contribute significantly to the diffraction pattern and suggests that a front-loaded pulse is preferred for imaging purposes. A previous study where a nonlocal thermodynamic equilibrium (nLTE) model was used to simulate the diffraction signal from protein nanocrystals came to a similar conclusion.^33^

The gating effect is seen when considering both ionization and atomic motion independently. When atoms are kept fixed in space but allowed to become ionized (purple, dashed lines in Fig. 6), the signal-to-noise accumulation follows a similar trend regardless of pulse duration. Values at pulse termination also show little variation for a given intensity. This is not all that surprising considering that the driver behind the gating effect in this case is the lowered scattering cross section due to electron depletion. Even with different exposure times, a constant number of photons applied to an immobile sample will generate a similar number of ionization events. The rate of scattering will vary, but the end states will be comparable. Lysozyme consists of 1960 atoms with composition C_613_H_959_N_193_O_185_S_10_, and our simulations predict the average ionization by the end of the pulse to be 1.00 and 1.45 per atom for the 5 fs and 50 fs pulses with 10^12^ photons, respectively. The difference mainly stems from Auger decay yet to happen and the increased X-ray transparency of photoionized atoms in the short-pulse case, but it does not seem to affect the SNR notably. Pulse intensity, on the other hand, has a considerable impact on expected signal-to-noise. Increasing the number of photons by one order of magnitude enhances the SNR accumulation approximately by a factor of 2 for all pulse durations. It seems obvious since the atomic positions are unchanged—additional photons will contribute to the signal, regardless of when they arrive, as long as there are electrons left to instigate scattering. As such, if ionization was the lone damaging process in SPI, pulse duration would be next to meaningless, while boosting the photon number would be beneficial up to the point where the sample is completely devoid of electrons.

Unfortunately, the resulting Coulomb forces provoke the ionic movement that appears to be the main SNR damage bottleneck at high resolutions. Displaced atoms continue to scatter, adding the signal to the pattern from spatial positions divergent from the initial structure, thus masking the true signal we are trying to measure. The green, dashed lines in Fig. 6 show how the displacement alone affects the SNR during exposure. It is clear that a shorter pulse allows for more diffraction signal to be recorded from the initial structure before the onset of the Coulomb explosion and therefore gives a higher signal-to-noise ratio. However, once movement has started to manifest the previously mentioned masking adds to the noise and a subsequent drop in SNR is observed. At the saturation point, the signal is no longer being washed out and SNR remains constant for the rest of the measurement. As noted earlier, the data from the longest pulses show that the saturation point is reached around *t* = 0 when half of the photons still remain, suggesting that the saturation is not an effect of the pulse reaching its termination. Instead, the gating must be a consequence of the damage processes, which potentially could provide an approach to SPI with longer pulses.

Our physical description of ionization dynamics has two major weaknesses, which at first may appear to make our results applicable only to the rather small proteins we have simulated. The first approximation is that we assume that the ejected free electrons do not cause any secondary electron impact ionization. Simulation studies have shown that in a neutral organic solid, a single 8 keV photoelectron can cause up to 400 secondary ionization events, and the Auger electrons cause cascades of tenths of electrons.^34^ However, for small systems like the ones studied here, most of these secondary ionizations will not occur since the electron mean free path is longer or on the same order as the size of the sample. The diameter of lysozyme is less than 10 nm. The radius of gyration of an electron cloud created from an 8 keV electron in an organic solid is on the order of 100 nm, and for a 250 eV carbon Auger electron, it is on the order of 10 nm.^19^ To relate our simulations to proteins larger than lysozyme, we compared the ionization in our system to those of an infinite lysozyme crystal. To do so we used an nLTE code, CRETIN.^28^ The average ionization at the end of each pulse from the nLTE simulations and our MD simulations is presented in Table I. In the 10^13^ photon case, the difference in the final average ionization between the MD simulations and the nLTE simulations is small. This is due to the fact that the direct photoionization is the dominating ionization pathway. This agreement indicates that our MD simulation accurately describes the ionization at these pulse intensities and that a larger molecule would follow a similar ionization rate. For the lower intensity, 10^12^ photons per pulse, we cannot draw the same conclusion, and the results we show are limited to samples of the size of lysozyme or smaller. The fact that the ionization is similar in the two models at high intensities is due to the fact that the ionization is governed predominately by the photo-ionization and the Auger decay. These two ionization channels are described similarly in the two models. At these high ionization states, effective electron impact ionization cross sections are different from those in a neutral sample, and this ionization channel only plays a minor role.

**TABLE I.** Average ionization of all atoms in lysozyme at the end of the X-ray pulse. Values from simulations of a single molecule using MD and a protein crystal using nLTE.

	10^12^ photons			10^13^ photons		
	5 fs	25 fs	50 fs	5 fs	25 fs	50 fs
MD	1.3	1.4	1.5	2.8	3.2	3.3
nLTE	3.3	3.4	3.5	3.5	3.5	3.5

Additionally, disregarding the treatment of free electrons inevitably leads to another indirect approximation in the diffraction calculations. Liberated electrons would contribute to Compton scattering, which is not accounted for here. However, we argue that this contribution would be comparably small. The free electrons that do remain within the sample would be highly energetic, and hence, we would expect them to be uniformly distributed. As a consequence, the resulting scattering would be at small angles and coincide with the central speckle. It is therefore unlikely that they would cause significant noise.

The second major approximation that is embedded in our model is the fact that we do not include any screening of the ions due to free electrons. Screening would be significant in a larger sample and would slow down the Coulomb explosion. Excluding screening means that we have a faster and more violent explosion in our simulations, which we expect would cause high noise in the diffracted signal. In other words, our model presents a worst-case scenario. Still, our SNR analysis shows that the sample inhomogeneity impairs SNR-levels to a greater extent than the explosion does.

It has recently been shown that the random orientation and the low photon signal per shot can be tackled with de novo correlation approaches and that a low number of coherently scattered photons are sufficient.^35^ Recent studies discuss ways to experimentally preorient a protein before the X-ray exposure,^36^ or orient the sample postexposure.^8^ The present study does not address the problem with finding the orientation of the sample as it is hit by the XFEL pulse but investigates the impact of damage on the diffraction patterns from aligned molecules. This has bearing on the feasibility of the orientation problem. In a sense, we are a taking a step toward a unified treatment of damage and the orientation problem.

## IV. CONCLUSIONS

There is little doubt that shorter X-ray pulses are superior to longer pulses for X-ray imaging of single biomolecules, especially if the intensity is high. However, it remains a profound challenge to experimentally generate sub-10 fs pulses with 10^12^ or more photons at the X-ray energies needed to achieve high resolution. While further developments toward these optimized pulses definitely are worth pursuing, the question remains if they really are necessary to achieve atomic resolution imaging.

By simulating the Coulomb explosion using molecular dynamics, we showed that the recorded diffraction patterns contain speckle information consistent with the initial structure. In fact, speckles from lysozyme seem more sensitive to natural structural variations than to the radiation damage induced from pulses up to 50 fs. Shot-to-shot differences due to damage are also significantly smaller than those caused by shot noise. These are all findings suggesting that damage may not be as detrimental as previously thought. Instead, focus should be put on reducing the structural variations of the sample between independent exposures. This can be achieved by incorporating techniques that are standard within mass spectrometry, such as sorting the sample based on conformation, mass, and charge.^37^ Furthermore, simulations have indicated that the structural stability of a protein in the gas phase can be enhanced by keeping some residual water on the sample,^38,39^ but there might be a need to find new ways to reduce the sample inhomogeneity.

Our data show that shot noise dominates damage noise over the full resolution span. The drastic loss in the signal-to-noise ratio induced by the former far exceeds the impact due to damage and should be of primary concern. This is the case for all pulse parameters studied, but the total effects on the SNR vary between them. As mentioned above, a short (5 fs) and intense (10^13^ photons) pulse optimizes the measured diffraction patterns. Yet interestingly, when employing longer pulses, a simultaneous decrease in intensity is also beneficial.

Finally, we found that both the ionization and atomic displacement components of the Coulomb explosion contribute to a gating effect of the diffraction. The damage processes onset by early photons lower the scattering cross sections, due to the loss of bound electrons around the atoms, and increase diffuse scattering, due to the displacement of atoms during exposure. This results in a saturation of the SNR and can be viewed equivalently to as if the sample where to experience a briefer pulse. This gives hope to the use of longer pulses in SPI, especially if the temporal profile of the pulse could be shaped to maximize the number of early arriving photons.

## References

[1] H. M. Berman , John Westbrook , Zukang Feng , Gary Gilliland , T. N. Bhat , Helge Weissig , Ilya N. Shindyalov , and P. E. Bourne , Nucl. Acids Res. 28, 235 (2000). 10.1093/nar/28.1.235 10592235 PMC102472

[2] X. Li , P. Mooney , S. Zheng , C. R. Booth , M. B. Braunfeld , S. Gubbens , D. A. Agard , and Y. Cheng , Nat. Methods 10, 584 (2013). 10.1038/nmeth.2472 23644547 PMC3684049

[3] X-C. Bai , I. S. Fernandez , G. McMullan , and S. H. Scheres , eLife 2, e00461 (2013). 10.7554/eLife.00461 23427024 PMC3576727

[4] M. Liao , E. Cao , D. Julius , and Y. Cheng , Nature 504, 107 (2013). 10.1038/nature12822 24305160 PMC4078027

[5] M. Khoshouei , R. Danev , J. M. Plitzko , and W. Baumeister , J. Mol. Biol. 429, 2611 (2017). 10.1016/j.jmb.2017.07.004 28697886

[6] R. Neutze , R. Wouts , D. van der Spoel , E. Weckert , and J. Hajdu , Nature 406, 752 (2000). 10.1038/35021099 10963603

[7] Z. Jurek , G. Oszlányi , and G. Faigel , Europhys. Lett. 65, 491 (2004). 10.1209/epl/i2003-10119-x

[8] C. Östlin , N. Tîmneanu , H. O. Jönsson , T. Ekeberg , A. V. Martin , and C. Caleman , Phys. Chem. Chem. Phys. 20, 12381 (2018). 10.1039/C7CP07267H 29488514

[9] H. N. Chapman , P. Fromme , A. Barty , T. A. White , R. A. Kirian , A. Aquila , M. S. Hunter , J. Schulz , D. P. DePonte , U. Weierstall , R. B. Doak , F. R. N. C. Maia , A. V. Martin , I. Schlichting , L. Lomb , N. Coppola , R. L. Shoeman , S. W. Epp , R. Hartmann , D. Rolles , A. Rudenko , L. Foucar , N. Kimmel , G. Weidenspointner , P. Holl , M. Liang , M. Barthelmess , C. Caleman , S. Boutet , M. J. Bogan , J. Krzywinski , C. Bostedt , S. Bajt , L. Gumprecht , B. Rudek , B. Erk , C. Schmidt , A. Hömke , C. Reich , D. Pietschner , L. Strüder , G. Hauser , H. Gorke , J. Ullrich , S. Herrmann , G. Schaller , F. Schopper , H. Soltau , K.-U. Kühnel , M. Messerschmidt , J. D. Bozek , S. P. Hau-Riege , M. Frank , C. Y. Hampton , R. G. Sierra , D. Starodub , G. J. Williams , J. Hajdu , N. Timneanu , M. M. Seibert , J. Andreasson , A. Rocker , O. Jönsson , M. Svenda , S. Stern , K. Nass , R. Andritschke , C.-D. Schröter , F. Krasniqi , M. Bott , K. E. Schmidt , X. Wang , I. Grotjohann , J. M. Holton , T. R. M. Barends , R. Neutze , S. Marchesini , R. Fromme , S. Schorb , D. Rupp , M. Adolph , T. Gorkhover , I. Andersson , H. Hirsemann , G. Potdevin , H. Graafsma , B. Nilsson , and J. C. H. Spence , Nature 470, 73 (2011). 10.1038/nature09750 21293373 PMC3429598

[10] A. Barty , C. Caleman , A. Aquila , N. Timneanu , L. Lomb , T. A. White , J. Andreasson , D. Arnlund , S. Bajt , T. R. M. Barends , M. Barthelmess , M. J. Bogan , C. Bostedt , J. D. Bozek , R. Coffee , N. Coppola , J. Davidsson , D. P. DePonte , R. B. Doak , T. Ekeberg , V. Elser , S. W. Epp , B. Erk , H. Fleckenstein , L. Foucar , P. Fromme , H. Graafsma , L. Gumprecht , J. Hajdu , C. Y. Hampton , R. Hartmann , A. Hartmann , G. Hauser , H. Hirsemann , P. Holl , M. S. Hunter , L. Johansson , S. Kassemeyer , N. Kimmel , R. A. Kirian , M. Liang , F. R. N. C. Maia , E. Malmerberg , S. Marchesini , A. V. Martin , K. Nass , R. Neutze , C. Reich , D. Rolles , B. Rudek , A. Rudenko , H. Scott , I. Schlichting , J. Schulz , M. M. Seibert , R. L. Shoeman , R. G. Sierra , H. Soltau , J. C. H. Spence , F. Stellato , S. Stern , L. Strüder , J. Ullrich , X. Wang , G. Weidenspointner , U. Weierstall , C. B. Wunderer , and H. N. Chapman , Nat. Photonics 6, 35 (2012). 10.1038/nphoton.2011.297 24078834 PMC3783007

[11] L. Galli , S.-K. Son , M. Klinge , S. Bajt , A. Barty , R. Bean , C. Betzel , K. R. Beyerlein , C. Caleman , R. B. Doak , M. Duszenko , H. Fleckenstein , C. Gati , B. Hunt , R. A. Kirian , M. Liang , M. H. Nanao , K. Nass , D. Oberthür , L. Redecke , R. Shoeman , F. Stellato , C. H. Yoon , T. A. White , O. Yefanov , J. Spence , and H. N. Chapman , Struct. Dyn. 2, 041703 (2015). 10.1063/1.4919398 26798803 PMC4711609

[12] K. Nass , L. Foucar , T. R. M. Barends , E. Hartmann , S. Botha , R. L. Shoeman , R. B. Doak , R. Alonso-Mori , A. Aquila , S. Bajt , A. Barty , R. Bean , K. R. Beyerlein , M. Bublitz , N. Drachmann , J. Gregersen , H. O. Jönsson , W. Kabsch , S. Kassemeyer , J. E. Koglin , M. Krumrey , D. Mattle , M. Messerschmidt , P. Nissen , L. Reinhard , O. Sitsel , D. Sokaras , G. J. Williams , S. Hau-Riege , N. Timneanu , C. Caleman , H. N. Chapman , S. Boutet , and I. Schlichting , J. Synchrotron Radiat. 22, 225 (2015). 10.1107/S1600577515002349 25723924

[13] S. P. Hau-Riege , R. A. London , G. Huldt , and H. N. Chapman , Phys. Rev. E 71, 061919 (2005). 10.1103/PhysRevE.71.061919 16089777

[14] M. W. Guetg , A. A. Lutman , Y. Ding , T. J. Maxwell , F.-J. Decker , U. Bergmann , and Z. Huang , Phys. Rev. Lett. 120, 014801 (2018). 10.1103/PhysRevLett.120.014801 29350964

[15] A. Aquila , A. Barty , C. Bostedt , S. Boutet , G. Carini , D. dePonte , P. Drell , S. Doniach , K. H. Downing , T. Earnest , H. Elmlund , V. Elser , M. Gühr , J. Hajdu , J. Hastings , S. P. Hau-Riege , Z. Huang , E. E. Lattman , F. R. N. C. Maia , S. Marchesini , A. Ourmazd , C. Pellegrini , R. Santra , I. Schlichting , C. Schroer , J. C. H. Spence , I. A. Vartanyants , S. Wakatsuki , W. I. Weis , and G. J. Williams , Struct. Dyn. 2, 041701 (2015). 10.1063/1.4918726 26798801 PMC4711616

[16] C. Caleman , N. Tîmneanu , A. V. Martin , H. O. Jönsson , A. Aquila , A. Barty , H. A. Scott , T. A. White , and H. N. Chapman , Opt. Express 23, 1213 (2015). 10.1364/OE.23.001213 25835880

[17] A. V. Martin , J. K. Corso , C. Caleman , N. Timneanu , and H. M. Quiney , IUCrJ 2, 661 (2015). 10.1107/S2052252515016887 PMC4645111 26594374

[18] N.-T. D. Loh and V. Elser , Phys. Rev. E 80, 026705 (2009). 10.1103/PhysRevE.80.026705 19792279

[19] C. Caleman , G. Huldt , F. R. N. C. Maia , C. Ortiz , F. G. Parak , J. Hajdu , D. van der Spoel , H. N. Chapman , and N. Timneanu , ACS Nano 5, 139 (2011). 10.1021/nn1020693 21138321

[20] C. Caleman , M. Bergh , H. A. Scott , J. C. Spence , H. N. Chapman , and N. Tîmneanu , J. Mod. Opt. 58, 1486 (2011). 10.1080/09500340.2011.597519

[21] K. Harata , Acta Crystallogr. D 50, 250 (1994). 10.1107/S0907444993013290 15299435

[22] E. Lindahl , B. Hess , and D. van der Spoel , J. Mol. Model. 7, 306 (2001). 10.1007/s008940100045

[23] B. F. Murphy , T. Osipov , Z. Jurek , L. Fang , S.-K. Son , M. Mucke , J. Eland , V. Zhaunerchyk , R. Feifel , L. Avaldi , P. Bolognesi , C. Bostedt , J. D. Bozek , J. Grilj , M. Guehr , L. J. Frasinski , J. Glownia , D. T. Ha , K. Hoffmann , E. Kukk , B. K. McFarland , C. Miron , E. Sistrunk , R. J. Squibb , K. Ueda , R. Santra , and N. Berrah , Nat. Commun. 5, 4281 (2014). 10.1038/ncomms5281 24969734

[24] C. Fortmann-Grote , A. Buzmakov , Z. Jurek , N.-T. D. Loh , L. Samoylova , R. Santra , E. A. Schneidmiller , T. Tschentscher , S. Yakubov , C. H. Yoon , M. V. Yurkov , B. Ziaja-Motyka , and A. P. Mancuso , IUCrJ 4, 560 (2017). 10.1107/S2052252517009496 PMC5619849 28989713

[25] W. L. Jorgensen and J. Tirado-Rives , J. Am. Chem. Soc. 110, 1657 (1988). 10.1021/ja00214a001 27557051

[26] M. Liang , G. J. Williams , M. Messerschmidt , M. M. Seibert , P. A. Montanez , M. Hayes , D. Milathianaki , A. Aquila , M. S. Hunter , J. E. Koglin , D. W. Schafer , S. Guillet , A. Busse , R. Bergan , W. Olson , K. Fox , N. Stewart , R. Curtis , A. A. Miahnahri , and S. Boutet , J. Synchrotron Radiat. 22, 514 (2015). 10.1107/S160057751500449X 25931062 PMC4416669

[27] R. Neutze , G. Huldt , J. Hajdu , and D. van der Spoel , Radiat. Phys. Chem. 71, 905 (2004). 10.1016/j.radphyschem.2004.04.121

[28] H. A. Scott , J. Quant. Spectrosc. Radiat. Transfer 71, 689 (2001). 10.1016/S0022-4073(01)00109-1

[29] K. R. Beyerlein , H. O. Jönsson , R. Alonso-Mori , A. Aquila , S. Bajt , A. Barty , R. Bean , J. E. Koglin , M. Messerschmidt , D. Ragazzon , D. Sokaras , G. J. Williams , S. Hau-Riege , S. Boutet , H. N. Chapman , N. Tîmneanu , and C. Caleman , Proc. Natl. Acad. Sci. 115, 5652 (2018). 10.1073/pnas.1711220115 29760050 PMC5984484

[30] H. O. Jönsson , C. Östlin , H. A. Scott , H. N. Chapman , S. J. Aplin , N. Tîmneanu , and C. Caleman , High Energy Density Phys. 26, 93 (2018). 10.1016/j.hedp.2018.02.004

[31] J. C. Slater , Phys. Rev. 36, 57 (1930). 10.1103/PhysRev.36.57

[32] M. Tegze and G. Bortel , J. Struct. Biol. 179, 41 (2012). 10.1016/j.jsb.2012.04.014 22575364

[33] H. O. Jönsson , N. Tîmneanu , C. Östlin , H. A. Scott , and C. Caleman , J. Synchrotron Radiat. 22, 256 (2015). 10.1107/S1600577515002878 25723927

[34] C. Caleman , C. Ortiz , E. Marklund , F. Bultmark , M. Gabrysch , F. G. Parak , J. Hajdu , M. Klintenberg , and N. Tîmneanu , Europhys. Lett. 85, 18005 (2009). 10.1209/0295-5075/85/18005

[35] B. van Ardenne , M. Mechelke , and H. Grubmüller , Nat. Commun. 9, 2375 (2018). 10.1038/s41467-018-04830-4 29915244 PMC6006178

[36] E. G. Marklund , T. Ekeberg , M. Moog , J. L. P. Benesch , and C. Caleman , J. Phys. Chem. Lett. 8, 4540 (2017). 10.1021/acs.jpclett.7b02005 28862456

[37] T. Wyttenbach , N. A. Pierson , D. E. Clemmer , and M. T. Bowers , Annu. Rev. Phys. Chem. 65, 175 (2014). 10.1146/annurev-physchem-040513-103644 24328447

[38] A. Patriksson , E. Marklund , and D. van der Spoel , Biochemistry 46, 933 (2007). 10.1021/bi061182y 17240977

[39] E. G. Marklund , D. S. D. Larsson , D. van der Spoel , A. Patriksson , and C. Caleman , Phys. Chem. Chem. Phys. 11, 8069 (2009). 10.1039/b903846a 19727514

